# Biomarkers of Sepsis

**DOI:** 10.3390/diagnostics13030435

**Published:** 2023-01-25

**Authors:** Luisa Agnello, Marcello Ciaccio

**Affiliations:** 1 Department of Biomedicine, Neurosciences and Advanced Diagnostics, Institute of Clinical Biochemistry, Clinical Molecular Medicine and Clinical Laboratory Medicine, University of Palermo, 90127 Palermo, Italy; 2 Department of Laboratory Medicine, University Hospital “P. Giaccone”, 90127 Palermo, Italy

Sepsis is a highly complex disease caused by a deregulated host’s response to infection. Recently, sepsis has been recognized as a global health priority by the World Health Organization due to its high mortality and morbidity. Rapidly detecting sepsis is crucial to prevent adverse outcomes and reduce mortality by promptly starting treatment. Indeed, it has been estimated that each hour of treatment delay is associated with a 7–10% increase in sepsis-related mortality. 

However, the early diagnosis of sepsis remains challenging because no specific signs and symptoms characterize it. Thus, many efforts have been made to identify reliable biomarkers for screening patients at high risk of sepsis. Additionally, sepsis biomarkers could provide information on prognosis and guide and monitor therapy. Thus, sepsis biomarkers represent a precious tool for clinical decision-making and for improving patient management.

In the last decade, the value of cell blood count (CBC) parameters as biomarkers for screening sepsis has emerged [1]. Beyond the basic CBC parameters, new cell population data provided by the new-generation hemocytometers displayed promising results as biomarkers of sepsis. Among these, monocyte distribution width (MDW) is the most investigated [2,3]. Agnello et al. have developed and validated an index, namely the Sepsis Index, based on the combination of MDW and the mean volume of monocytes by a mathematical formula [4,5]. It showed better performance than the single biomarkers, reaching a sensitivity and specificity of 80% and 91%, respectively, for detecting sepsis. Noteworthily, the Sepsis Index showed a negative predictive value of 98%. Thus, a normal value can exclude sepsis with high accuracy. 

Red cell distribution width (RDW), which indicates the anisocytosis of erythrocytes, is another promising biomarker of sepsis. Wang et al. [6] have recently shown that RDW has a good prognostic value for sepsis. Indeed, increased RDW was associated with an increased risk of 30-day in-hospital mortality, septic shock, and ICU admission. Additionally, RDW was an independent predictor of overall mortality.

Another easy-to-measure biomarker of sepsis is glucose. Hung et al. showed that blood glucose levels measured on the first day of hospitalization could guide mortality risk stratification in critically ill patients with sepsis [7]. 

To date, the prognostic performance of several biomarkers has been investigated, achieving mixed findings. 

Chen et al. assessed the diagnostic and prognostic value of pentraxin-3 (PTX3) in patients with sepsis and septic shock admitted to the ICU [8]. Patients with septic shock had the highest levels of PTX3. Additionally, it was an independent predictor of mortality in sepsis and septic shock patients. Thus, PTX3 could be a reliable prognostic biomarker of sepsis and septic shock. 

Yagmur et al. first explored the possible role of clusterin as a prognostic biomarker in critically ill patients admitted to ICU, with and without sepsis [9]. Clusterin is ubiquitously expressed and plays pleiotropic functions, including regulating the immune system. The authors showed increased levels of clusterin in patients than in healthy controls. However, it was not associated with disease severity, organ failure, or mortality. Thus, further studies are mandatory to elucidate the usefulness of clusterin in critically ill patients. 

Interestingly, Kyriakoudi et al. evaluated the possible role of IL-18 and its binding protein (IL-18BP) as biomarkers of weaning outcomes in patients with sepsis [10]. They showed that constantly high levels of IL-18 and IL-18BP were indicative of spontaneous breathing trial failure in patients recovering from sepsis. These findings encourage further research on the predictive role of IL-18/ and IL-18BP for SBT outcomes in patients with sepsis. Programmed death protein (PD-1) is another inflammatory marker proposed as a diagnostic and prognostic tool for sepsis [11]. It is a checkpoint inhibitor factor expressed on several immune system cells, including activated T lymphocytes, NK cells, B lymphocytes, and monocytes. PD-1 levels are increased in patients with sepsis, and it may play an important role in regulating T lymphocytes and monocytes during sepsis. Sari et al. exhaustively described the evidence on PD-1 and its ligand, PD-L1, as biomarkers of sepsis [11]. 

Finally, an interesting field of research is the study of non-codingRNA, including long non-codingRNAs (lncRNAs), circular RNAs (circRNAs), and microRNAs (miRNAs). Di Raimondo et al. summarized the literature evidence on circRNA-miRNA-lncRNA networks as sepsis biomarkers and therapeutic targets focusing on their clinical usefulness [12]. 

Sepsis is a life-threatening disease and early diagnosis and appropriate management are crucial to reducing its mortality and morbidity. Several potential sepsis biomarkers involved in different pathological mechanisms have been assessed. In addition, recent advances in technology have led to the development of innovative and promising biomarkers such as the non-coding RNAs. However, most of these biomarkers did not pass all the steps to be introduced into clinical practice. 

Sepsis is a very heterogeneous disease and there is still a long way to go in finding the ideal biomarker.
